# Liposomal versus standard bupivacaine for post-operative opioid requirements following abdominal-based breast reconstruction: A systematic review and meta-analysis

**DOI:** 10.1016/j.jpra.2026.02.025

**Published:** 2026-02-28

**Authors:** Guilherme Franceschini Machado, Jonathan Mokhtar, Victoria Trasatti Romão, Alexandre G. Lellouch

**Affiliations:** a Hospital San Paolo, São Paulo, Brazil; b Mohammed Bin Rashid University of Medicine and Health Sciences, College of Medicine (MBRU-CoM), Dubai, United Arab Emirates; c Faculdade Anhembi Morumbi, São Paulo, Brazil; d Division of Plastic and Reconstructive Surgery, Cedars Sinai Hospital, Los Angeles, USA; e Université Paris Cité, Inserm The Paris Cardiovascular Research Center, Team Endotheliopathy and Hemostasis Disorders, Paris, France; f 2- AP-HP, Hôpital Européen Georges Pompidou, Hematology department, Paris, France

**Keywords:** Abdominal-based breast reconstruction, Postoperative analgesia, Transverse abdominus plane (TAP) block, Liposomal bupivacaine, Enhanced recovery after surgery (ERAS)

## Abstract

**Background:**

Donor-site pain after abdominal-based autologous breast reconstruction affects opioid use and recovery. Liposomal bupivacaine (LB) delivered via the transversus abdominis plane (TAP) block may enhance analgesia compared with standard bupivacaine (SB), yet its added clinical benefit remains unclear.

**Methods:**

A systematic review and meta-analysis was conducted according to PRISMA 2020 and Cochrane guidelines. Databases searched included PubMed, Embase, and Cochrane Library. Studies comparing LB with SB for the TAP block were included. Outcomes included postoperative opioid consumption, pain scores, hospital length of stay (LOS), time to ambulation, and complications. Both pairwise and single-arm meta-analyses were performed using a random-effects model.

**Results:**

A total of twelve studies were included in the analysis, comprising 1,690 participants. Pairwise meta-analyses indicated a trend favoring the use of LB in relation to total opioid use (SMD, –0.17; 95% CI [–1.20, 0.86]; *p* = 0.75) and LOS (SMD, –0.83 days; 95% CI [–1.76, 0.09]; *p* = 0.08). However, neither finding was statistically significant. In single-arm analyses, the pooled inpatient opioid use with LB was 132.61 oral morphine equivalents (OME), with post-anesthesia care unit (PACU) use at 17.56 OME and an average LOS of 3.39 days. The pooled rates of seroma and flap loss were 8% and 1.08%, respectively. Additionally, cost and ambulation outcomes varied across the studies.

**Conclusion:**

Liposomal bupivacaine appears safe and may offer modest recovery benefits in abdominal-based breast reconstruction. However, its incremental advantage over standard bupivacaine remains uncertain in ERAS-optimized settings. Selective use is recommended pending further high-quality trials.

## Introduction

Postoperative pain following abdominal-based flap breast reconstruction is a significant clinical concern and a primary driver of opioid consumption.^1^ Opioid use is higher in bilateral reconstruction, prolonged operations, and in patients with psychiatric comorbidities such as anxiety and depression.^2^^,^^3^ This main issue has been further exacerbated by the opioid epidemic in the United States, which peaked around 2016 with an estimated 65,000 opioid-related deaths that year.^4^ In response to the growing need for opioid-sparing strategies, especially among elderly patients who are vulnerable to opioid-related events in autologous breast reconstructive surgery,^5^ transversus abdominis plane (TAP) blocks with liposomal bupivacaine (LB) have emerged as a significant component of multimodal analgesia, particularly within Enhanced Recovery After Surgery (ERAS) protocols.

TAP block targets the anterior abdominal wall and has demonstrated effectiveness in reducing acute postoperative pain and opioid requirements in a range of bariatric, colorectal, and breast procedures.^6^ Among the local anesthetics used in breast reconstructive surgery, an extended long-acting LB has gained interest due to its potential to prolong analgesia beyond the first 24 hours (h) postoperatively.^7^^,^^8^ The reduction of postoperative opioid requirements, lower pain scores beyond the first 24 h, and shorter hospital stays following the use of LB in TAP blocks are usually pronounced in subgroups of unilateral or delayed breast reconstruction.^9^^,^^10^ While LB is generally safe and well tolerated, the consistency of its clinical benefit when compared to standard bupivacaine (SB) remains uncertain and not clinically meaningful.^11^^,^^12^ Previous meta-analyses have grouped LB with a broad array of other anesthetic agents, such as ropivacaine and levobupivacaine (chirocaine), used across anatomical sites and analgesic techniques. This broader inclusion could underscore the ability to draw conclusions related particularly to the deep inferior epigastric artery perforator (DIEP) flap or abdominal-based breast reconstruction, where the TAP block is the most relevant and consistently used technique.^11^^,^^13^^,^^14^ As a result, the true comparative effectiveness of LB to SB should be adequately evaluated.

To address this gap, we conducted a systematic review and meta-analysis to evaluate the effectiveness and quantify the clinically relevant effects of LB versus SB when used via TAP block in patients undergoing autologous abdominal-based breast reconstruction. Our primary objective was to assess differences in opioid consumption, pain scores, length of hospital stay, and postoperative complication rates, such as seroma. We hypothesized that LB, when used as part of an ERAS-based multimodal strategy TAP block, could provide clinically significant reductions in opioid use or superior recovery outcomes compared to SB.

## Methods

This systematic review with meta-analysis was registered in the International Prospective Register of Systematic Reviews and Clinical Trials (PROSPERO) under the protocol ID CRD420251050107 (available online). The study was designed following the Cochrane Collaboration Handbook for Systematic Reviews of Interventions and the Preferred Reporting Items for Systematic Reviews and Meta-Analysis (PRISMA) Statement Guidelines **(Figure, Supplemental Digital Content 1).**^15^

### Eligibility criteria

Studies were eligible for inclusion if they met all of the following criteria: (1) enrolled adult patients (aged 18 years or older); (2) investigated individuals undergoing autologous breast reconstruction using abdominally based flaps, such as the DIEP flap, transverse rectus abdominus myocutaneous (TRAM) flap (free or pedicled), and muscle-sparing transverse rectus abdominus myocutaneous (MS-TRAM) flap; (3) administered either LB or SB for postoperative pain management; and (4) utilized the TAP block as the delivery technique for analgesia.

Exclusion criteria were applied to studies that: (1) included non-abdominal donor sites (e.g., latissimus dorsi, gluteal flaps); (2) implant-based breast reconstruction; (3) non-human subjects; (4) weren't original studies (e.g, review articles, editorials, letters, protocols, or conference abstracts); and (5) did not report relevant postoperative outcomes.

### Search strategy and data extraction

We systematically searched PubMed, Embase, and the Cochrane Library from inception to April 2025 with the following search terms: *(“liposomal”) AND (“bupivacaine”) AND (“transversus abdominis plane block” OR “TAP block” OR “deep inferior epigastric perforator flap” OR DIEP OR “breast reconstruction” OR “breast surgery” OR “transverse rectus abdominis myocutaneous” OR “transverse rectus abdominis musculocutaneous” OR “TRAM” OR “MS-TRAM” OR msTRAM OR “superficial inferior epigastric” OR SIEA OR “abdominally based free flap”)*. Additional references were manually searched to identify any missing articles that the search strategy hadn’t provided (snowballing references from previous literature).

Two authors (G.F.M. and V.T.R.) performed the screening using Rayyan (Rayyan Systems Inc., Cambridge, MA, USA). Baseline characteristics and outcome data were extracted from the included studies by two authors (J.M. and G.F.M.) using Microsoft Excel, and a third author (V.T.R.) independently verified the quality of the extracted data and removed any duplicate entries; any disagreements were resolved through consensus and consultation with the senior author.

### Endpoints and sub-analyses

#### Primary and secondary outcomes

This systematic review and meta-analysis evaluated clinical and recovery-related outcomes in patients undergoing abdominal-based autologous breast reconstruction with postoperative analgesia using LB or SB. Primary outcomes included postoperative opioid consumption (measured in morphine milligram equivalents [oral morphine equivalents (OME)]), pain scores at standardized postoperative intervals (24, 48, and 72 h), and length of hospital stay (LOS).

Secondary outcomes included postoperative complications (seroma, hematoma, cellulitis, and flap loss) and time to ambulation. Readmission rates and economic metrics (e.g., cost per patient) were qualitatively analyzed. When reported, post-anesthesia care unit (PACU) opioid use and inpatient opioid consumption were also extracted. Pain scores were assessed using validated instruments such as the Visual Analog Scale (VAS) or the Numerical Rating Scale (NRS), and outcomes were analyzed in both pairwise comparisons and single-arm summaries.

#### Subgroup analyses

Subgroup analyses were performed to examine pain scores stratified by postoperative time points (24, 48, and 72 h), aiming to assess whether the analgesic effects of LB varied over time. This approach enabled a time-sensitive evaluation of pain control, particularly in the early postoperative period, when analgesic demand is typically highest.

### Sensitivity analyses

A leave-one-out sensitivity analysis was conducted for outcomes with high reported heterogeneity (I^2^ > 40%). This methodical evaluation allowed for an assessment of the influence of individual studies on the overall results.

### Quality assessment

We assessed the methodological quality of the included studies using tools aligned with study design. For randomized controlled trials (RCTs), we used the Risk of Bias 2.0 (RoB 2.0) tool. For non-randomized studies, we applied the Risk Of Bias In Non-randomized Studies – of Interventions (ROBINS-I) tool. Two reviewers (G.F.M. and V.T.R.) independently assessed the risk of bias. Disagreements were resolved by discussion and consensus or by consulting a third reviewer (J.M.).

To assess the overall certainty of evidence for each outcome, we applied the GRADE^16^ (Grading of Recommendations Assessment, Development and Evaluation) framework. The GRADE approach was applied consistently across pairwise outcomes, and all judgments were reviewed collaboratively by the study team.

### Statistical analysis

Meta-analyses were conducted using a rigorous approach, applying a random-effects model to account for expected variation across studies, as implemented in R (version 4.3.3). Effect sizes were reported as standardized mean differences (SMDs) for continuous outcomes and odds ratios (ORs) with 95% confidence intervals (CIs) for dichotomous outcomes. Between-study heterogeneity was assessed using the I² statistic and Cochran’s Q test; I² values greater than 40% indicated substantial heterogeneity. For outcomes with low heterogeneity (I² ≤ 25%), a standard random-effects model using the DerSimonian and Laird (DL) method was employed. Sensitivity analyses were conducted using the generic inverse-variance method, incorporating adjusted effect estimates when available, to assess the stability of the pooled results and the impact of individual studies.

## Results

### Study selection and characteristics

**Figure 1:**
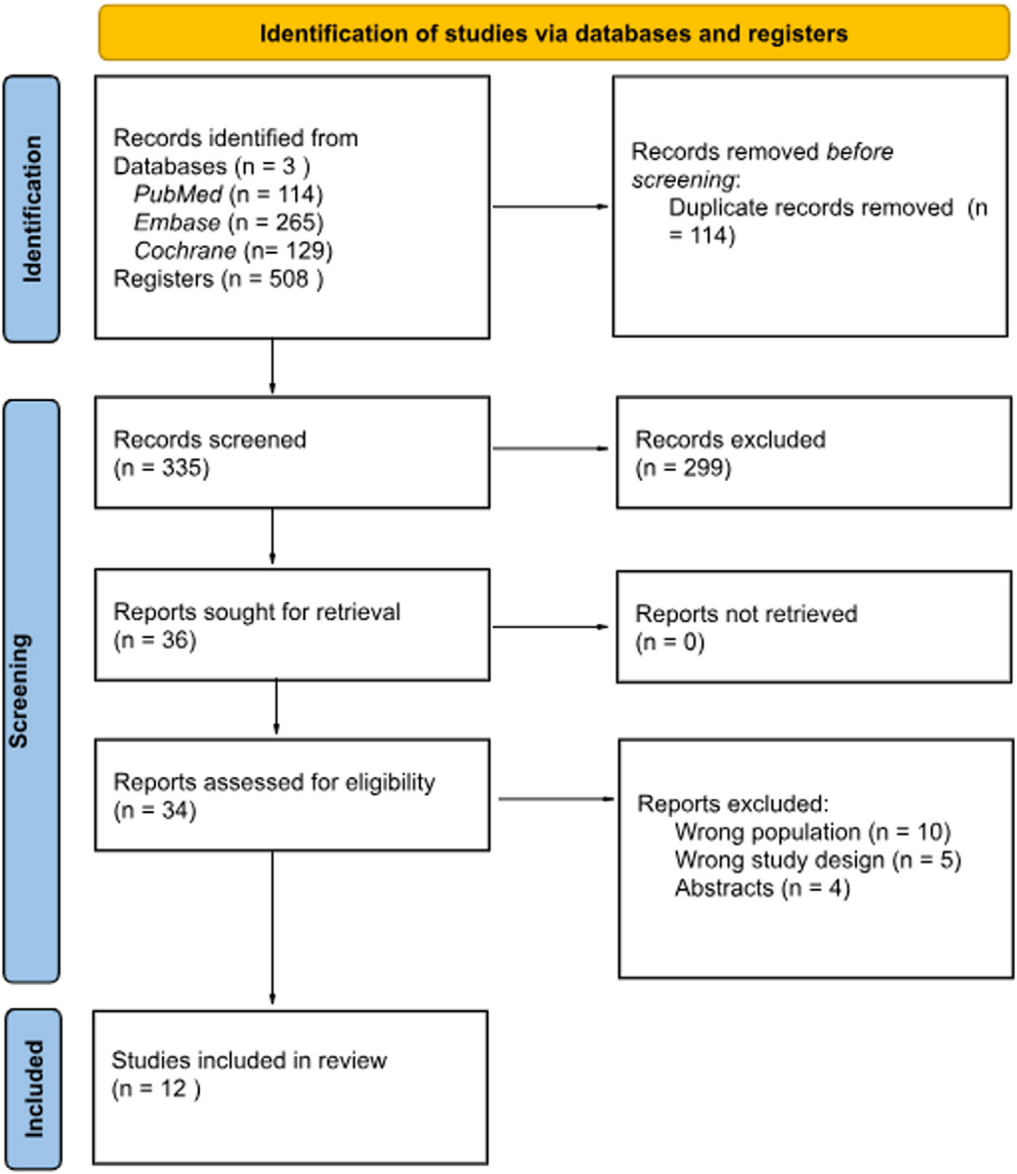
PRISMA flow diagram of study selection.

As outlined in Figure 1, the initial search identified 1,245 records. After the removal of duplicates and exclusion of irrelevant titles and abstracts (due to incorrect interventions, outcomes, or study designs), 46 full-text articles were reviewed for eligibility by two independent reviewers (G.F.M. and V.T.R.) using predefined inclusion criteria. Ultimately, 12 studies^7^^,^^9^^,^^10^^,^^14^^,^^17^, ^18^, ^19^, ^20^, ^21^, ^22^, ^23^, ^24^ met the eligibility requirements and were included in the meta-analysis. These studies encompassed a total of 1,690 patients who underwent abdominally based autologous breast reconstruction with postoperative analgesia using either LB or SB.

Both pairwise meta-analyses (comparing LB and SB) and single-arm analyses (reporting LB outcomes only) were conducted to evaluate primary and secondary endpoints. The characteristics of the included studies, including design type, sample size, flap type, analgesic approach, and radiotherapy, are detailed in Table 1.

**Table 1:** Baseline characteristics of included studies in this meta-analysis.

Study	Country	Study design	Groups	Sample (*n*)	Age (years)	BMI (kg/m^2^)	Unilateral DIEP (%)	Bilateral DIEP (%)	Immediate BR (%)	Delayed BR (%)	Chemotherapy (%)	Chest radiation (%)
Clary et al.^7^	USA	Retrospective cohort	LB	15	NS	NS	46.67	53.33	0	100	NS	NS
Gatherwright et al.^10^	USA	RCT	LB SB	8 8	52.1^a^ 53.1^a^	30^a^ 28^a^	100 100	0 0	0 0	100 100	NS	NS
Ha et al.^20^	USA	RCT	LB SB	22 22	49 ± 9.2^b^ 49 ± 10.0^b^	29.1 ± 4.6^b^ 28.1 ± 4.5^b^	36.4 36.4	63.6 63.6	13.64 22.72	86.36 77.28	77.3 77.3	54.5 68.2
Haddock et al.^17^	USA	Retrospective cohort	LB	80	52 ± 9.2^b^	30.1 ± 4.8^b^	15	85	66.3	33.8	30	25
Jablonka et al.^9^	USA	Retrospective cohort	LB	40	50.2 ± 8.5^b^	28 ± 5.4^b^	45	55	82.5	17.5	17.5	30
Knackstedt et al.^21^	USA	Retrospective cohort	LB SB	6 6	NS	NS	0 0	100 100	0 0	100 100	NS	NS
Knackstedt et al.^22^	USA	Retrospective cohort	LB SB	669 348	51.7 ± 9.8^b^ 50.4 ± 9.2^b^	NS	44.1 26.7	55.9 73.3	1	99 100	NS	NS
Lombana et al.^23^	USA	Retrospective cohort	LB SB	38 30	48.6 ± 9.8^b^ 49.3 ± 8.1^b^	30.5 ± 5.1^b^ 30.2 ± 4.3^b^	21 27	NS	39 27	61 73	32 57	71 50
Momeni et al.^18^	USA	Retrospective cohort	LB	46	47.6 ± 9.8^b^	28.1 ± 5.6^b^	NS	NS	55	45	NS	NS
Nguyen et al.^14^	USA	RCT	LB SB	30 30	53 ± 9.5^b^ 52.2 ± 9.8^b^	29.6 ± 5.3^b^ 30.2 ± 4.3^b^	30 23	70 77	17 20	83 80	33 33	20 33
Rendon et al.^19^	USA	Retrospective cohort	LB	39	54.9 ± 8.9^b^	31.3 ± 5.0^b^	49	51	64	36	NS	NS
Salibian et al.^24^	USA	Retrospective cohort	LB SB	50 64	49.17 ± 1.4^b^ 51.3 ± 1.0^b^	31.2 ± 0.8^b^ 30.2 ± 0.6^b^	38 39.1	62 60.9	74.1 74.8	25.9 25.2	32 18.8	18 15.6

Abbreviations: RCT, Randomized Controlled Trial, LB, Liposomal Bupivacaine, SB, Standard Bupivacaine, NS, Not Specified, BMI, Body Mass Index, BR, Breast Reconstruction.

a mean, mean ± standard deviation (sd).

b mean ± standard deviation (sd).

### Meta-analysis of primary and secondary outcomes

#### Inpatient opioid consumption – pairwise

**Figure 2:**
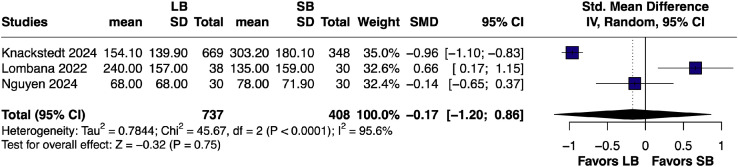
Forest plot of inpatient opioid consumption (pairwise analysis of LB vs SB).

Across three studies,^14^^,^^22^^,^^23^ the pooled SMD in inpatient opioid consumption favored lower consumption in the LB cohort over SB, though not significantly (SMD –0.17, 95% CI [–1.20, 0.86]; *p* = 0.75; I² = 95.6%; Figure 2).

#### Intravenous narcotic use – pairwise

**Figure 3:**
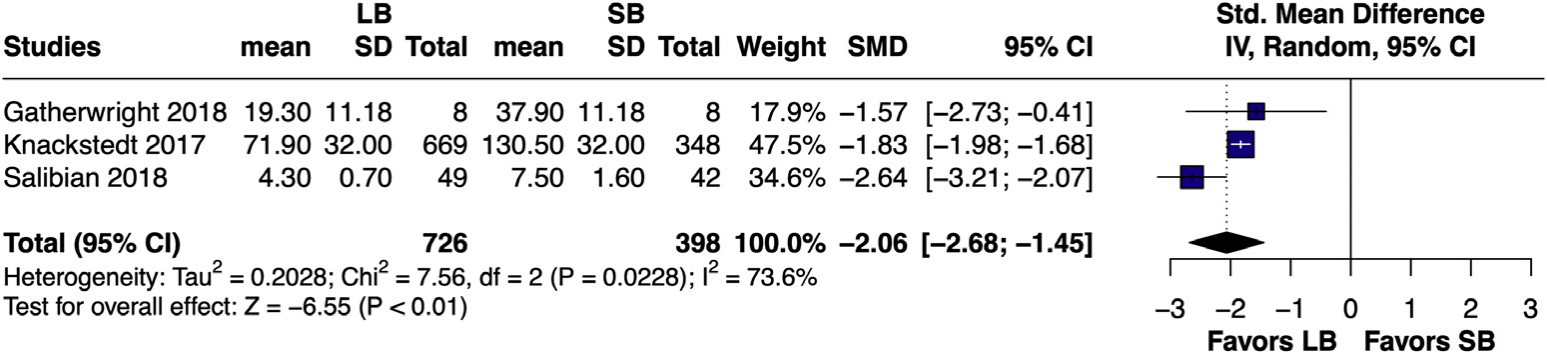
Forest plot of intravenous narcotic use (pairwise analysis of LB vs SB).

A random-effects meta-analysis comparing long-acting LB and short-acting SB intravenous narcotic use yielded an SMD of –2.06 (95% CI [–2.68, –1.45]; *p* < 0.01). This analysis demonstrates significant heterogeneity across studies^10^^,^^21^^,^^24^ (I² = 73.6%), highlighting considerable variability in the effect estimates (Figure 3).

#### Time-point pain scores (24 h, 48 h, 72 h)

**Figure 4:**
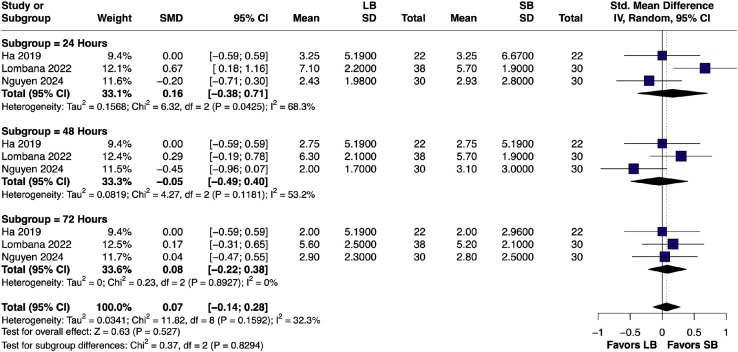
Forest plots of postoperative pain scores at 24 h, 48 h, and 72 h (pairwise analysis of LB vs SB).

At 24 h postoperatively the SMD in pain scores was 0.16 (95% CI [–0.38, 0.71]; I² = 68.3%), followed by –0.05 (95% CI [–0.49, 0.40]; I² = 53.2%) at 48 h, and 0.08 (95% CI [–0.22, 0.38]; I² = 0%) at 72 h. No significant differences were observed between studies^14^^,^^20^^,^^23^ at any postoperative interval (*p* = 0.83); however, heterogeneity decreased substantially by 72 h (Figure 4).

#### Seroma – pairwise

**Figure 5:**
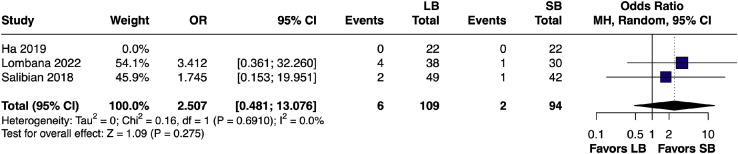
Forest plot of seroma incidence (pairwise analysis of LB vs SB).

The pooled OR for seroma formation showed no significant increase with LB^20^^,^^23^^,^^24^ (OR 2.51, 95% CI [0.48, 13.08]; I² = 0%), indicating equivalent safety compared to SB (Figure 5).

#### Total narcotic use – pairwise

**Figure 6:**
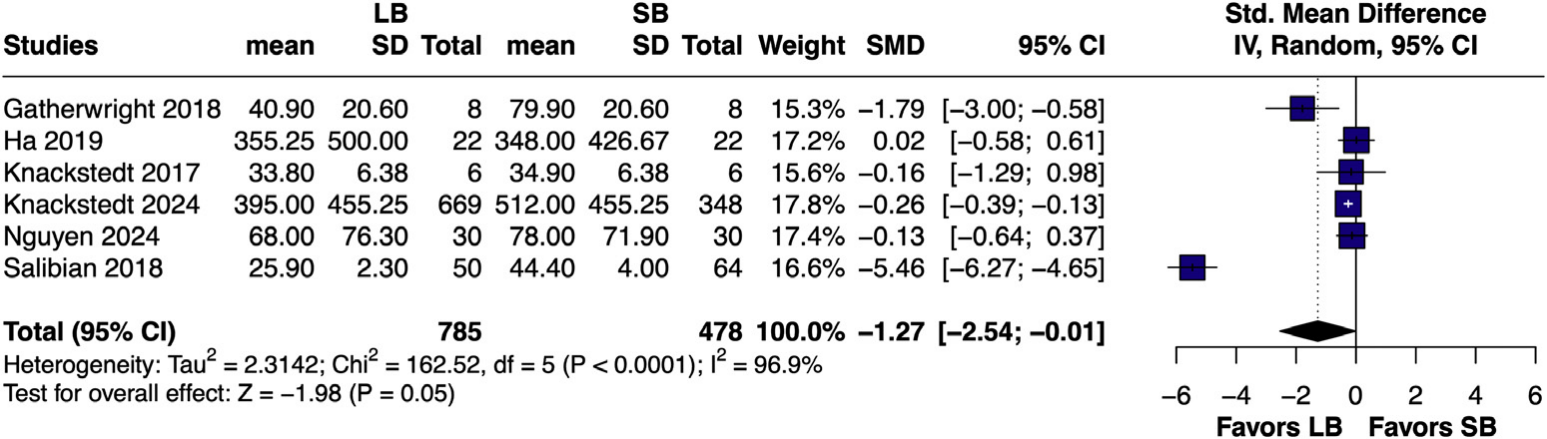
Forest plot of total narcotic use (pairwise analysis of LB vs SB).

The pooled SMD for total narcotic use^10^^,^^14^^,^^20^, ^21^, ^22^^,^^24^ was –1.27 (95% CI [–2.54, –0.01]; *p* = 0.05; I² = 96.9%), again favoring LB with a clinically significant trend (Figure 6).

#### Length of hospital stay – pairwise

**Figure 7:**
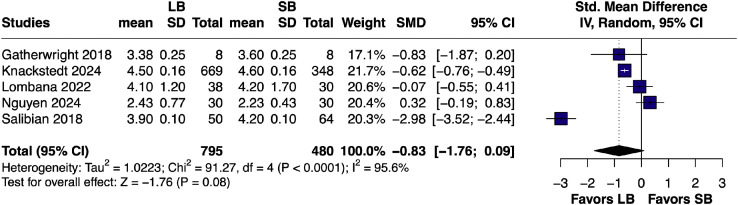
Forest plot of length of hospital stays (pairwise analysis of LB vs SB).

A random-effects meta-analysis of five studies^10^^,^^14^^,^^22^, ^23^, ^24^ comparing LB to SB for postoperative LOS showed a pooled SMD of -0.83 days (95% CI [–1.76, 0.09]: *p* = 0.08). Heterogeneity was high (I = 95.6%) (Figure 7).

#### Length of hospital stay – single arm

**Figure 8:**
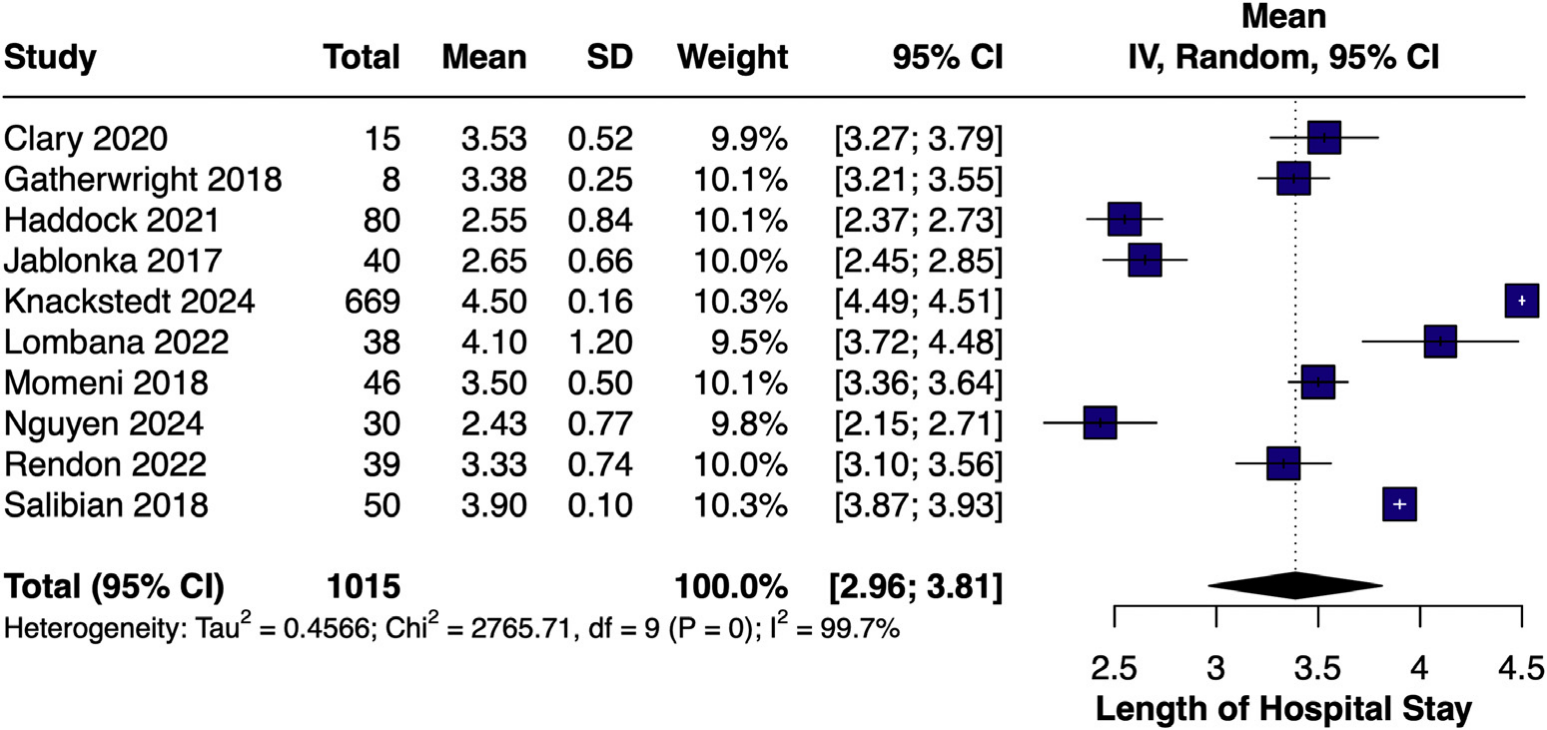
Forest plot of length of hospital stays (single-arm LB).

A single-arm meta-analysis comprising 10 studies^7^^,^^9^^,^^10^^,^^14^^,^^17^, ^18^, ^19^^,^^22^^,^^23^^,^^24^ and 1015 patients yielded a pooled mean hospital LOS of 3.39 days (95% CI [2.96, 3.81]; I² = 99.7%) (Figure 8).

#### Inpatient opioid consumption – single-arm

**Figure 9:**
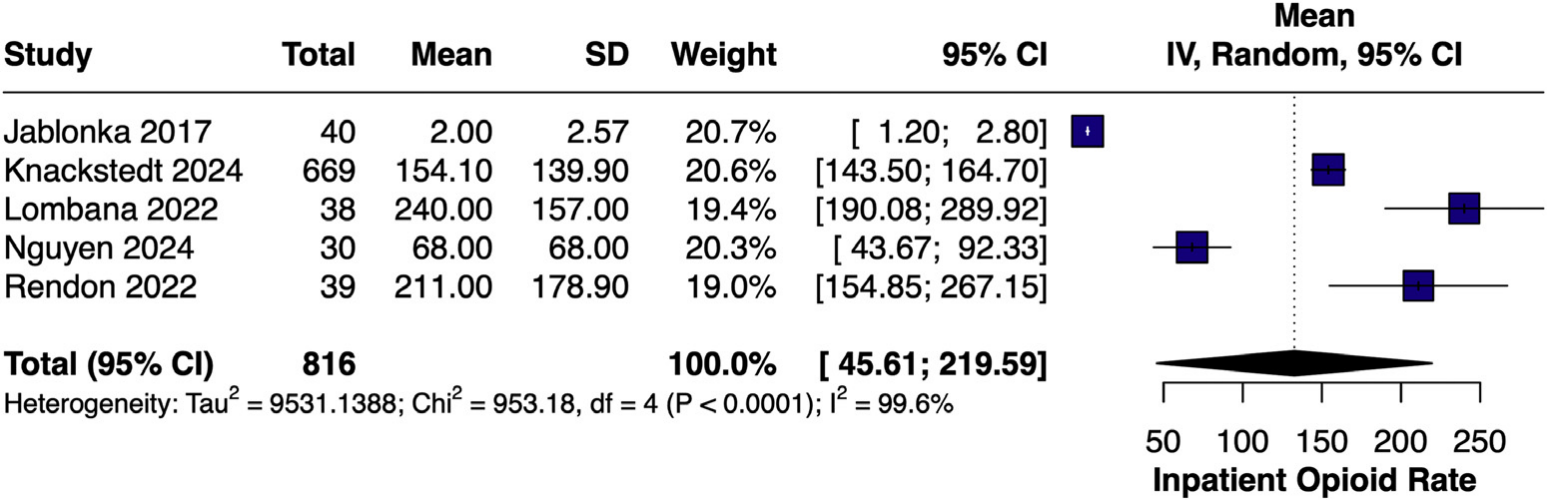
Forest plot of inpatient opioid consumption (single-arm LB).

The pooled mean inpatient opioid use for LB-only cohorts^9^^,^^14^^,^^19^^,^^22^^,^^23^ was 132.61 OME (95% CI [45.61, 219.59]; I² = 99.6%), reflecting substantial inter-study variability in dosing and reporting. (Figure 9).

#### PACU opioid use – single-arm

**Figure 10:**
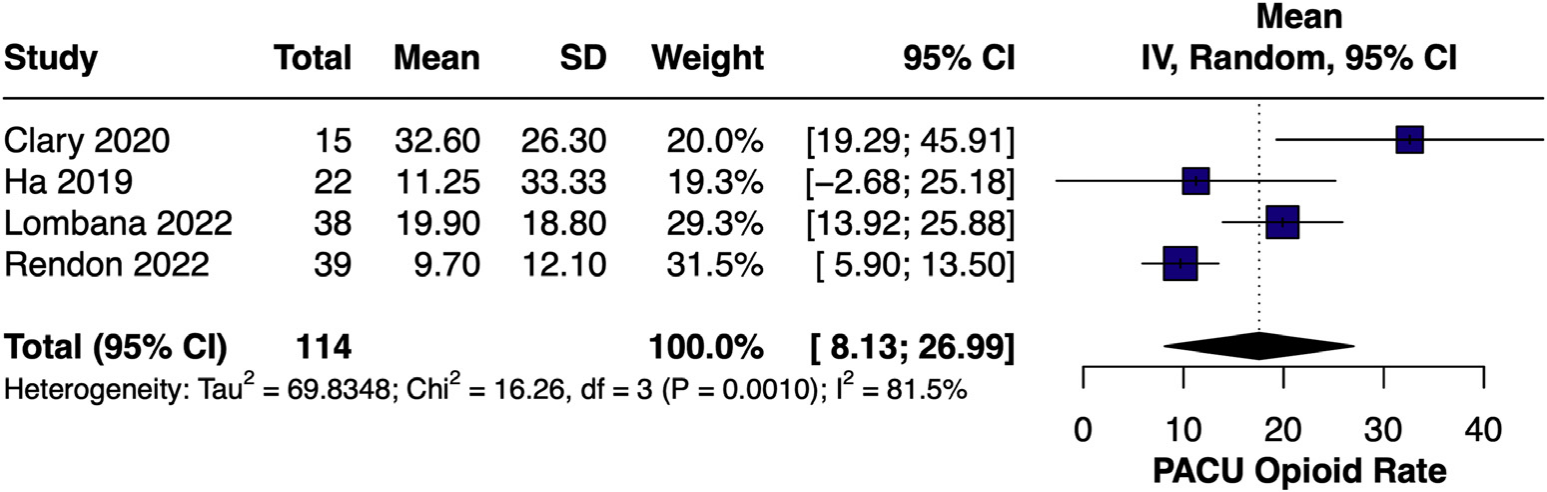
Forest plot of PACU opioid use (single-arm LB).

The pooled mean PACU opioid requirement^7^^,^^19^^,^^20^^,^^23^ was 17.56 OME (95% CI [8.13, 26.99]; I² = 81.5%), highlighting consistent early postoperative analgesic benefit with LB (Figure 10).

#### Seroma – single-arm

**Figure 11:**
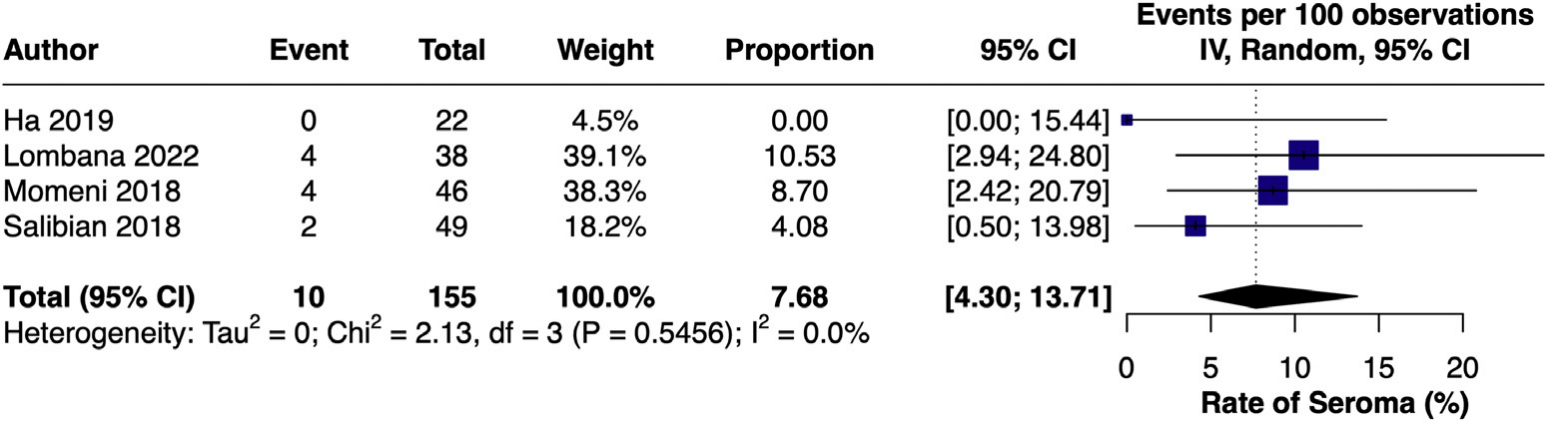
Forest plot of seroma incidence (single-arm LB).

The pooled incidence of seroma among LB-treated patients^18^^,^^20^^,^^23^^,^^24^ was 7.7% (95% CI [4.30, 13.71]; I² = 0.0%), indicating a low and stable complication rate across studies (Figure 11).

#### Flap loss – single arm

**Figure 12:**
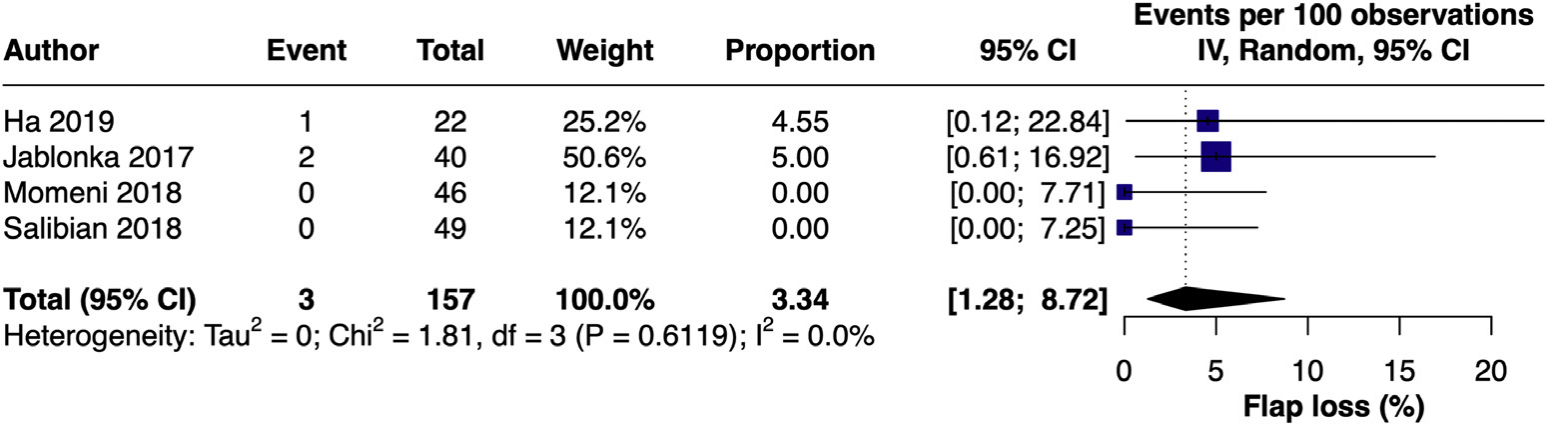
Forest plot of flap loss incidence (single-arm LB).

A single-arm meta-analysis of four studies^9^^,^^18^^,^^20^^,^^24^ assessing flap loss yielded a pooled incidence of 3.34% (95% CI [1.28, 8.72]) using a random-effects model with Freeman-Tukey double arcsine transformation. Heterogeneity was negligible (I² = 0.0%) Figure 12).

#### Total narcotic use – single-arm

**Figure 13:**
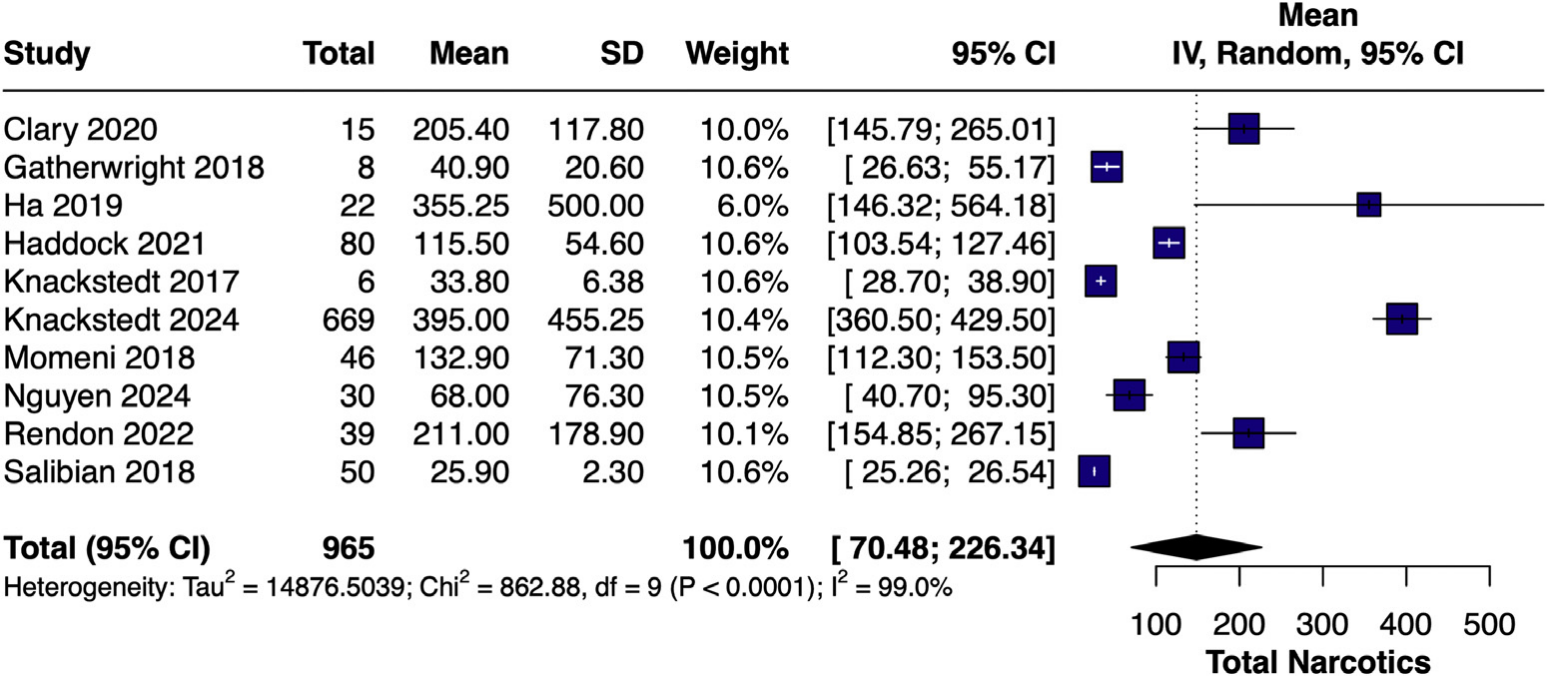
Forest plot of total narcotic use (single-arm LB).

The pooled average for total narcotic consumption across LB studies^7^^,^^10^^,^^14^^,^^17^, ^18^, ^19^, ^20^, ^21^, ^22^^,^^24^ was 135.44 OME (95% CI [70.48, 226.34]; I² = 99.0%), corroborating the findings from paired analyses (Figure 13).

### Sensitivity analysis

In the analysis of postoperative pain scores at 24 and 48 h, the exclusion of Lombana et al*.*^23^ yielded a marked reduction in heterogeneity from 68.3% to 0% at 24 h, and from 53.2% to 19.6% at 48 h **(Figures, Supplemental Digital Content 2 and 3)**. However, despite performing a leave-one-out sensitivity analysis across all remaining outcomes, heterogeneity remained largely unchanged **(Figures, Supplemental Digital Content 4 through 9)**. This persistence suggests that underlying differences in patient characteristics, surgical technique, perioperative protocols, or other unmeasured factors likely contributed to the observed variability.

### Quality assessment

**Figure 14:**
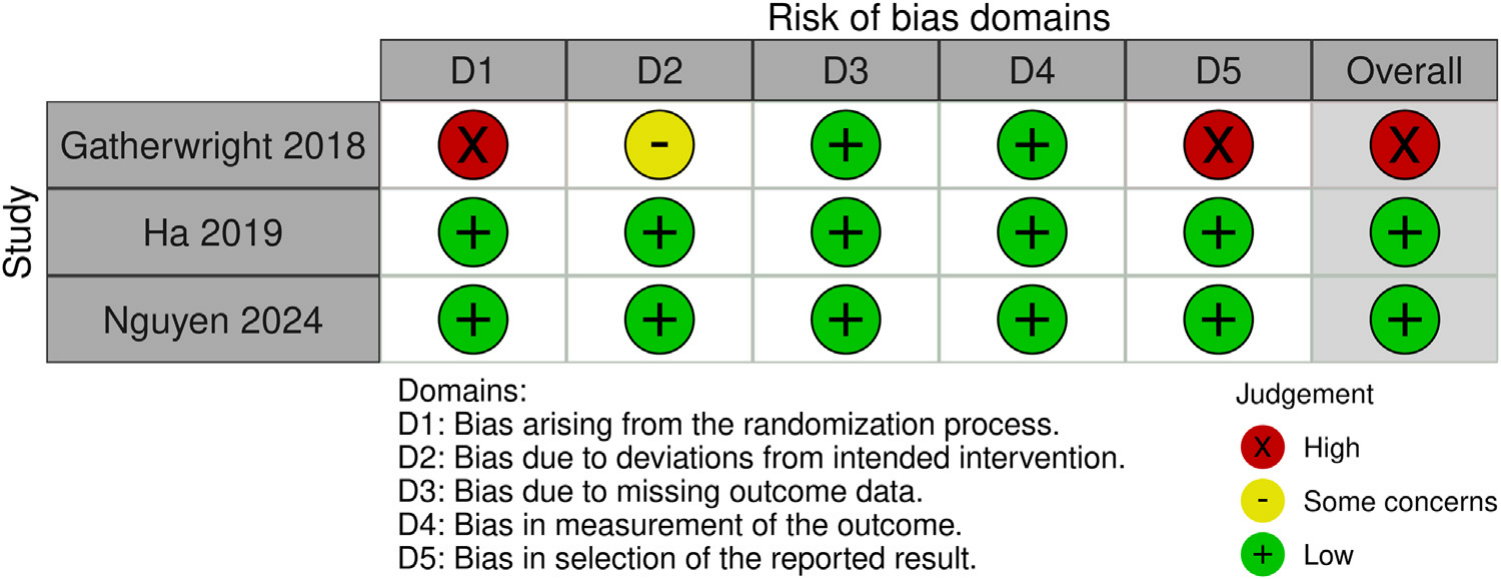
Risk of bias summary for randomized controlled trials.

**Figure 15:**
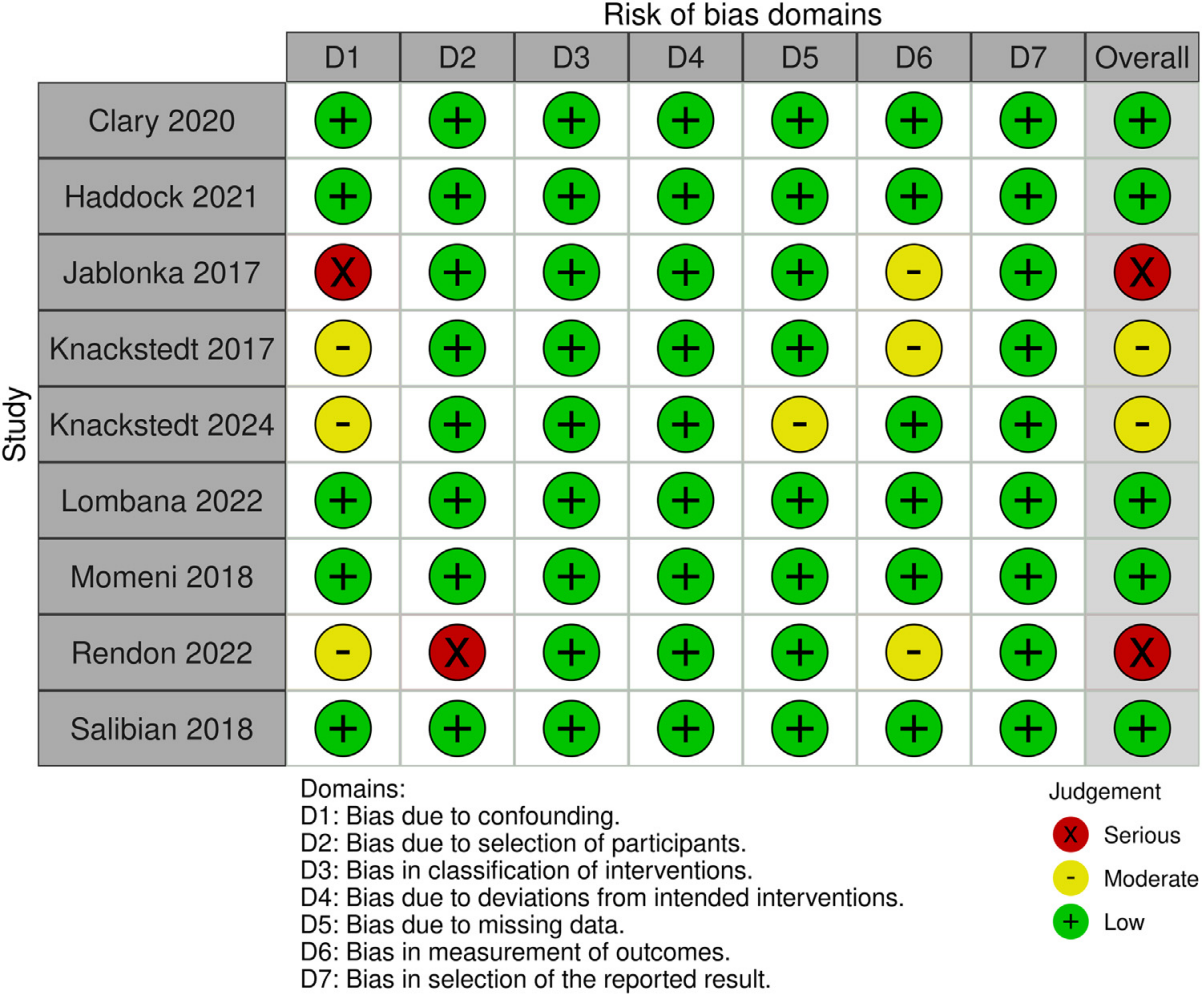
Risk of bias summary for observational studies.

Regarding three randomized trials, two were rated as low risk, while one study,^10^ was rated as high risk due to concerns regarding randomization and selective reporting. Among the nine observational studies, five showed a low risk, two moderate risk, and two serious risk of bias, mainly due to participant selection and the lack of statistical adjustment. (Figure 14, Figure 15).

### Certainty of evidence

The certainty of evidence ranged from very low to moderate. Outcomes such as inpatient opioid consumption, total opioid use, and length of stay were graded as very low certainty due to substantial heterogeneity (I² > 95%), reliance on non-randomized studies, and wide confidence intervals crossing the null effect. The outcome IV opioid use showed a large effect favoring LB, supported by consistent findings across studies, however, it was downgraded to low certainty due to risk of bias and moderate heterogeneity (I² = 73.6%). Pain scores at 24 and 48 h were graded as low certainty, while pain scores at 72 h were graded as moderate certainty due to consistency (I² = 0%) and more precise estimates.

## Discussion

In this systematic review and meta-analysis of 12 studies involving 1,690 patients undergoing autologous abdominal-based breast reconstruction, we evaluated the impact of LB compared to SB when delivered via a TAP block. LB may modestly reduce opioid consumption and pain scores in the first 24 h. However, these reductions were not consistently observed across all studies, especially those conducted within the ERAS protocol.^14^^,^^22^ Single-arm data suggested slightly more favorable outcomes in pain control and early mobilization, but pairwise comparison did not demonstrate statistical significance. Importantly, we found no consistent evidence of reduced complication rates or long-term adverse effects associated with LB.

This meta-analysis addresses a critical gap by focusing specifically on abdominal-based flap breast reconstruction, while clarifying that although LB offers pharmacologic advantages, it does not consistently demonstrate superior clinical outcomes when compared with SB. Several potential explanations may account for the limited incremental benefit of LB. Although designed for extended release, the effect of LB may be diminished when multimodal analgesia is already optimized. Variability in the TAP block technique, the individual performing the procedure, the injection location over time, and the dosage may also influence its analgesic efficacy. More potent effects reported in retrospective studies may reflect selection or reporting bias (Salibian et al.^24^*)*. In addition, pain originates from two distinct surgical fields, while TAP blocks address only abdominal wall innervation, limiting the interpretation of global pain scores.

Clinically, our findings suggest that routine use of LB in TAP blocks provides limited additional benefit over SB in ERAS-optimized settings. However, in institutions with less consistent ERAS adherence, LB may support enhanced recovery, reduced resource use, and improved patient comfort. Evidence on costs was mixed: two studies^10^^,^^22^ reported slightly higher costs with LB, whereas another study^24^ reported a $633 per-patient saving. These discrepancies likely reflect institutional variability in practices and cost definitions.

Despite heterogeneity in IV narcotic use, LOS plots, and included observational studies, this review represents the first meta-analysis focused specifically on LB versus SB in TAP blocks for abdominal-based breast reconstruction. By narrowing the clinical context, applying strict inclusion criteria, and analyzing pairwise and single-arm outcomes separately, it provides a clinically meaningful synthesis that balances efficacy and limitations to support decision-making.

## Limitations and future directions

This study is subject to several limitations that must be considered. First, the most significant limitation is the substantial heterogeneity across the included studies. This high variability is likely multifactorial, stemming from differences in patient selection, specific ERAS protocols, and the type or timing of the TAP block. However, the leave-one-out sensitivity analysis conducted in this meta-analysis accounted for studies that contributed to this heterogeneity, and effect sizes remained consistent despite their omission.

Second, most included studies were non-randomized, inherently increasing susceptibility to bias. The absence of blinding in many of these studies, particularly given the long-acting pharmacologic profile of LB raises the potential for performance and detection bias that may influence both patient-reported pain scores and the administration of rescue analgesia. Nevertheless, our methodological rigor, including a comprehensive risk-of-bias assessment and a GRADE-based evaluation of the certainty of the evidence, strengthens the validity and transparency of our overall findings.

Future research should include multicenter randomized trials with standardized TAP block protocols, with consistent timing (e.g., longer follow-ups for pain ≥7 days) and dosing. Pain should be categorized using validated tools by mechanism (e.g., the McGill Pain Questionnaire [MPQ]) and analyzed separately by surgical site. Analyses should be stratified by ERAS adherence to identify settings where LB offers benefit, and standardized cost-effectiveness studies using quality-adjusted life year (QALYs) are needed to assess economic impact across institutions.

## Conclusion

This meta-analysis showcases that liposomal bupivacaine is a safe component of multimodal analgesia in this patient population. However, given the lack of statistically significant superiority over standard bupivacaine and the substantial cost difference, its routine use cannot be universally recommended. Selective use of LB may be warranted in specific high-risk subgroups, such as patients with a history of chronic pain or opioid dependence, where any potential marginal benefit in opioid-sparing could be clinically meaningful. Future high-quality, adequately powered, randomized controlled trials are necessary to precisely define the role and cost-effectiveness of LB in modern, ERAS-optimized breast reconstruction pathways.

## Availability of data

Data is available upon reasonable request from the corresponding author.

## Declaration of competing interest

The authors declare no financial or commercial conflicts of interest related to the content of this manuscript.

## Appendix Supplementary materials

Figure, Supplementary Digital Content 1. PRISMA checklist. Image, application 1

Figure, Supplementary Digital Content 2. Sensitivity analysis of postoperative pain score at 24 h (pairwise meta-analysis). Image, application 2

Figure, Supplementary Digital Content 3. Sensitivity analysis of postoperative pain score at 48 h (pairwise meta-analysis). Image, application 3

Figure, Supplementary Digital Content 4. Sensitivity analysis of inpatient opioid consumption (pairwise meta-analysis). Image, application 4

Figure, Supplementary Digital Content 5. Sensitivity analysis of inpatient opioid consumption (single-arm meta-analysis). Image, application 5

Figure, Supplementary Digital Content 6. Sensitivity analysis of the length of hospital stays (pair-wise meta-analysis). Image, application 6

Figure, Supplementary Digital Content 7. Sensitivity analysis of the length of hospital stays (single-arm meta-analysis). Image, application 7

Figure, Supplementary Digital Content 8. Sensitivity analysis of total narcotic use (single-arm meta-analysis). Image, application 8

Figure, Supplementary Digital Content 9. Sensitivity analysis of PACU opioid consumption (single-arm meta-analysis). Image, application 9
