# Supplementary figures and images for: Liposomal versus standard bupivacaine for post-operative opioid requirements following abdominal-based breast reconstruction: A systematic review and meta-analysis
Source: JPRAS Open. 2026 Feb 28;49:253–66. doi: 10.1016/j.jpra.2026.02.025 (PMC13015253; doi:10.1016/j.jpra.2026.02.025)

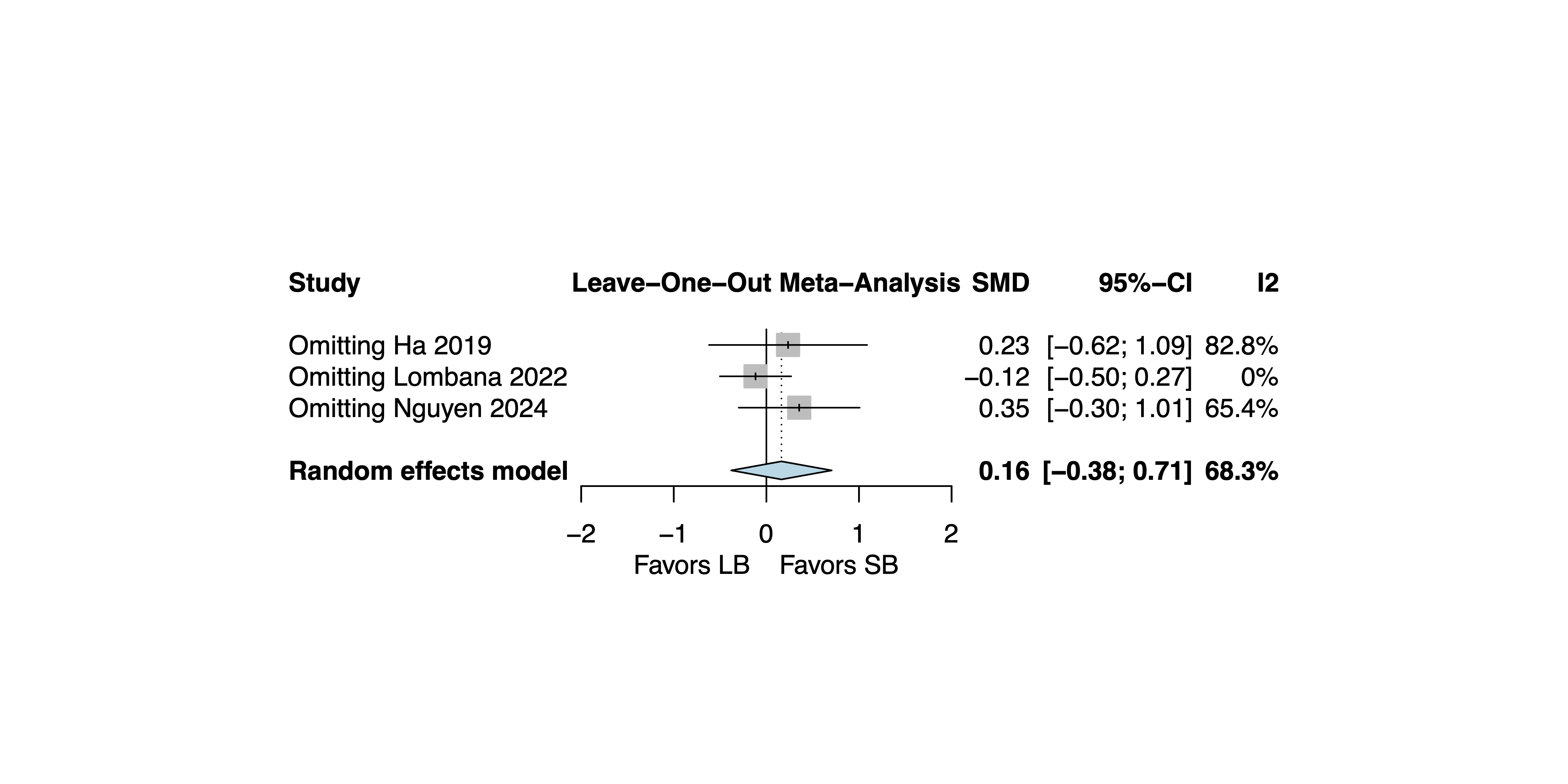

Supplement: Supplementary file 2 — Figure, Supplementary Digital Content 2. Sensitivity analysis of postoperative pain score at 24 h (pairwise meta-analysis). [file mmc2.zip › mmc2.png]

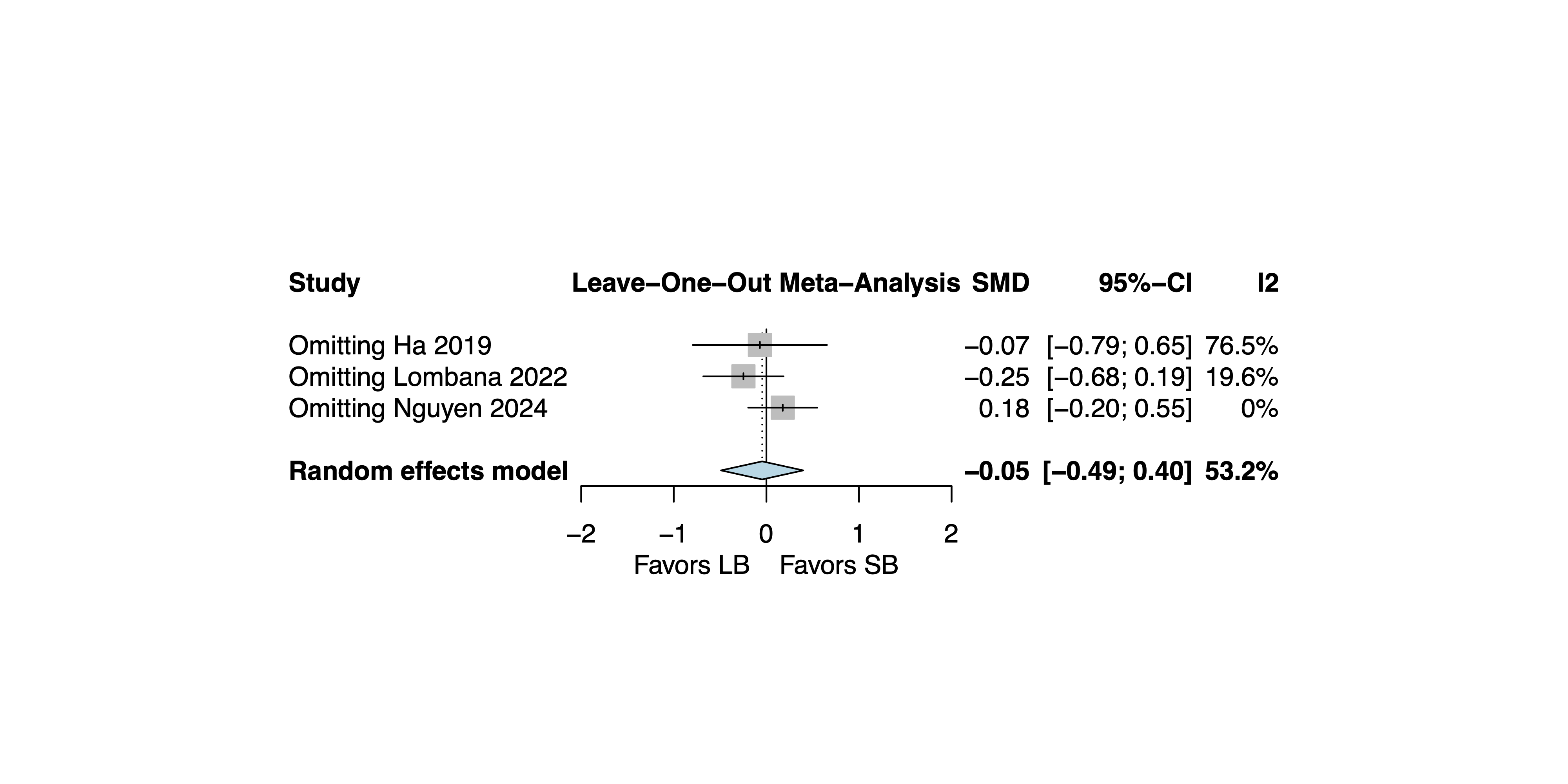

Supplement: Supplementary file 3 — Figure, Supplementary Digital Content 3. Sensitivity analysis of postoperative pain score at 48 h (pairwise meta-analysis). [file mmc3.zip › mmc3.png]

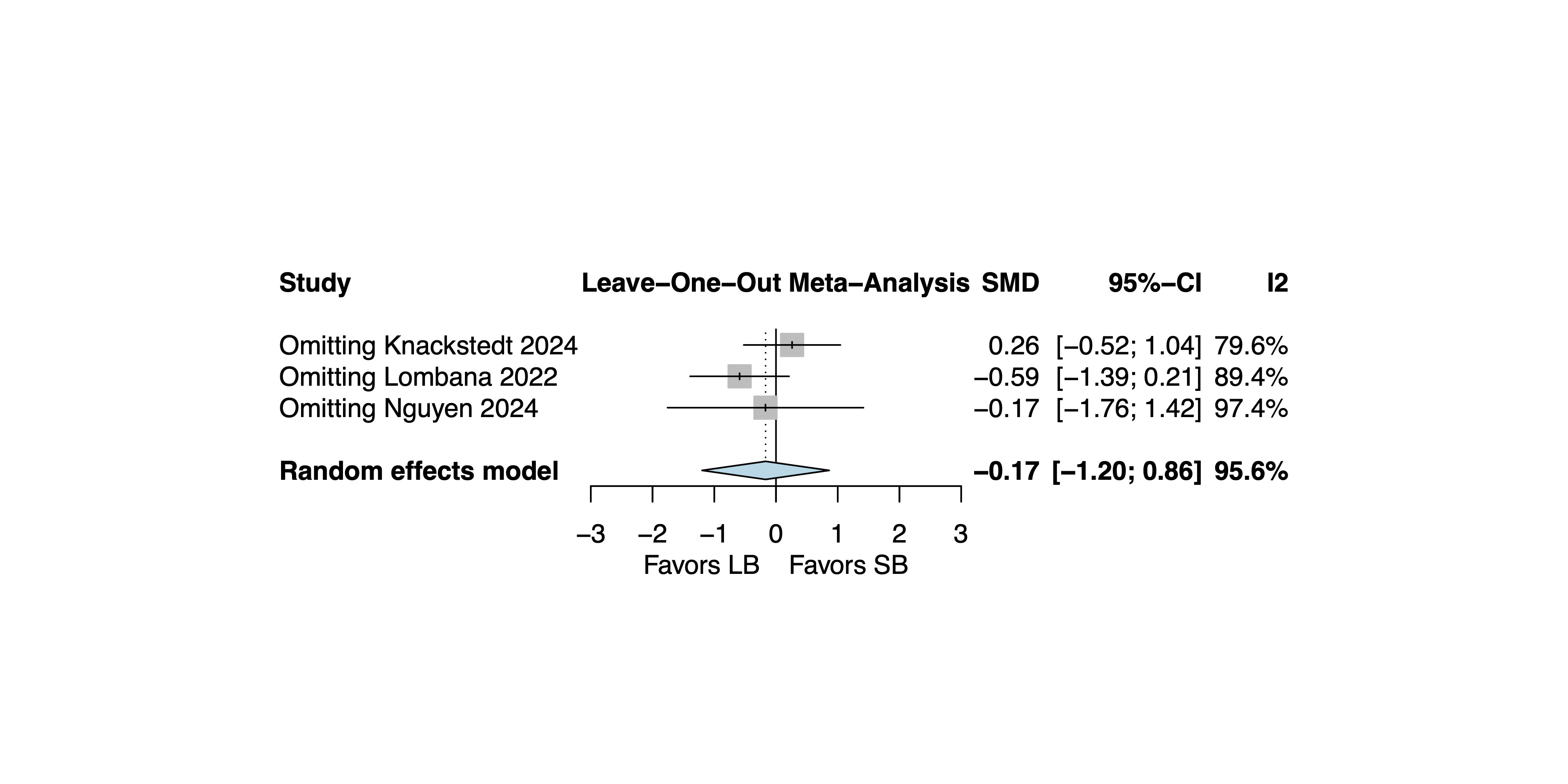

Supplement: Supplementary file 4 — Figure, Supplementary Digital Content 4. Sensitivity analysis of inpatient opioid consumption (pairwise meta-analysis). [file mmc4.zip › mmc4.png]

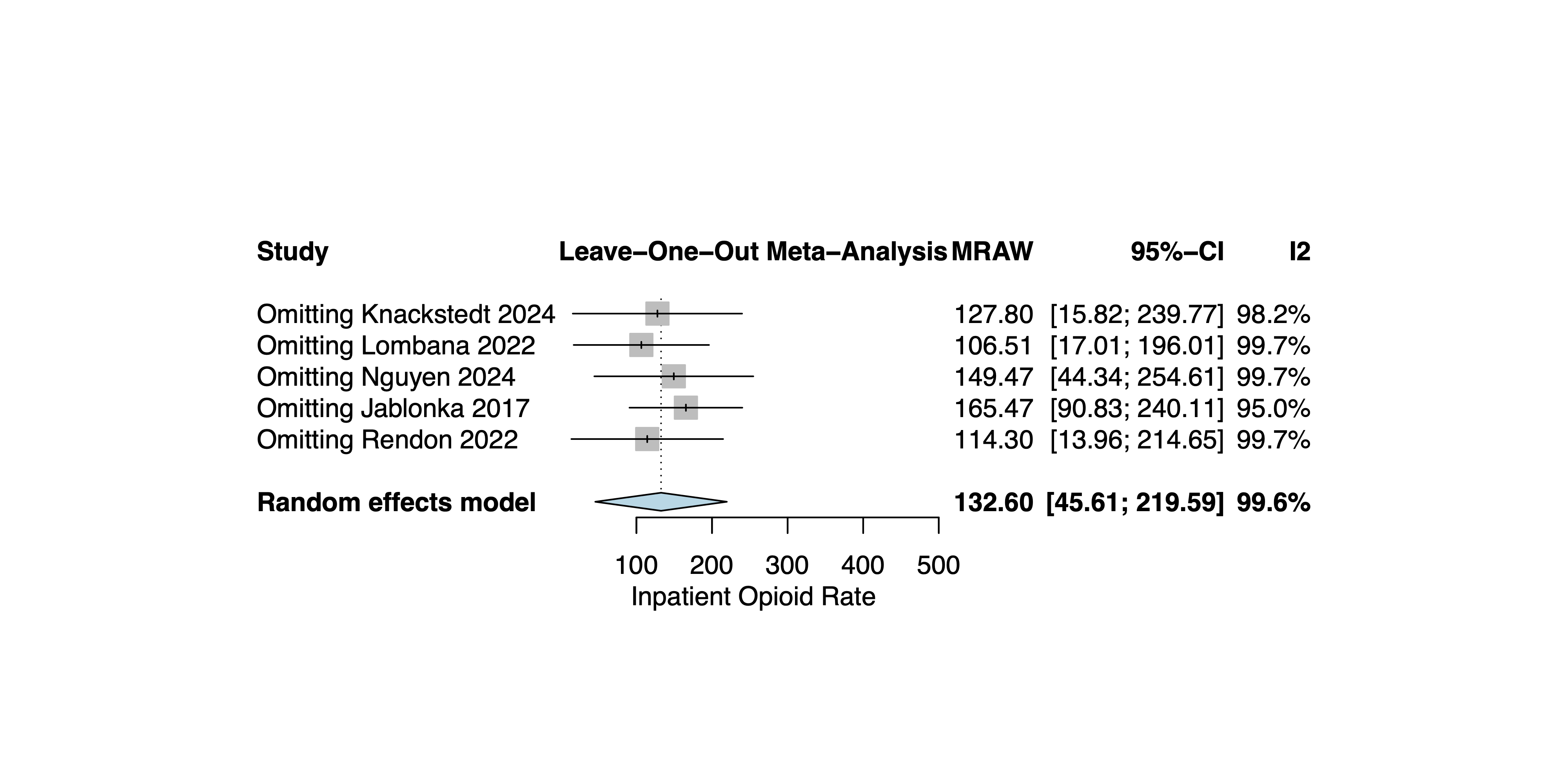

Supplement: Supplementary file 5 — Figure, Supplementary Digital Content 5. Sensitivity analysis of inpatient opioid consumption (single-arm meta-analysis). [file mmc5.zip › mmc5.png]

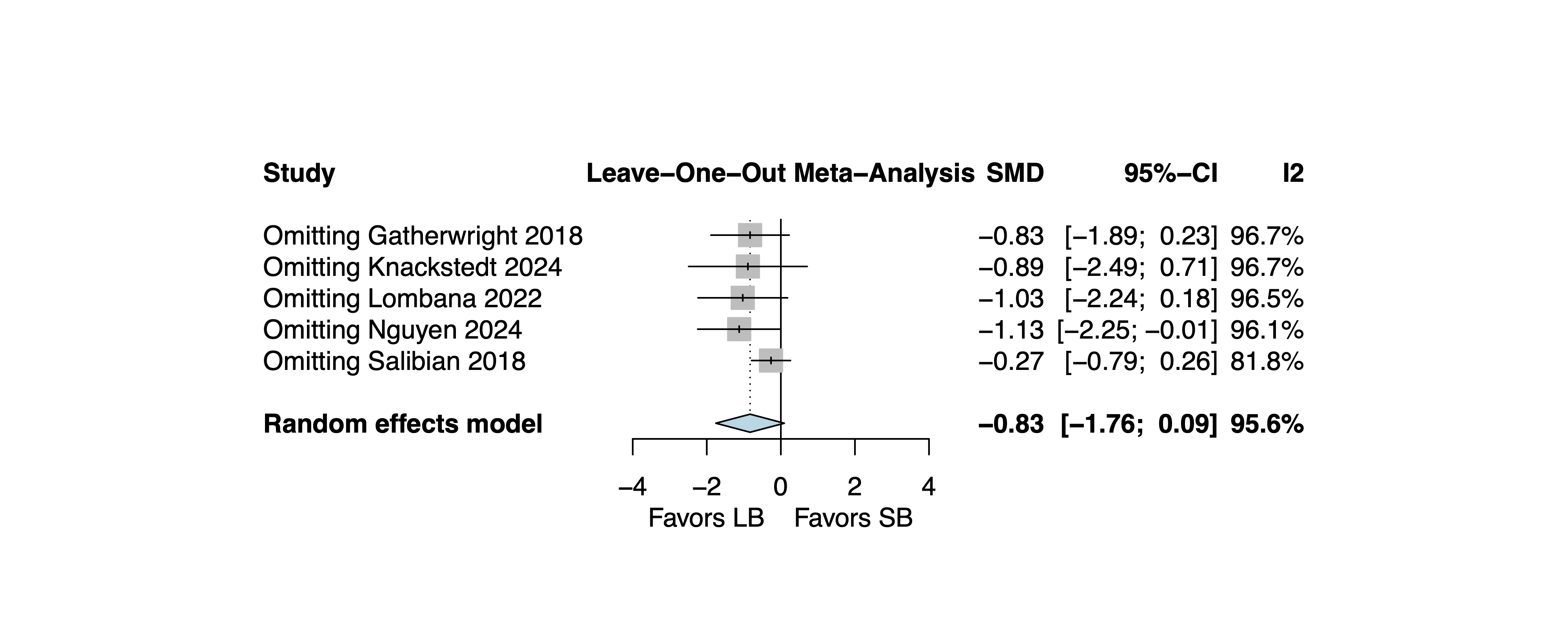

Supplement: Supplementary file 6 — Figure, Supplementary Digital Content 6. Sensitivity analysis of the length of hospital stays (pair-wise meta-analysis). [file mmc6.zip › mmc6.png]

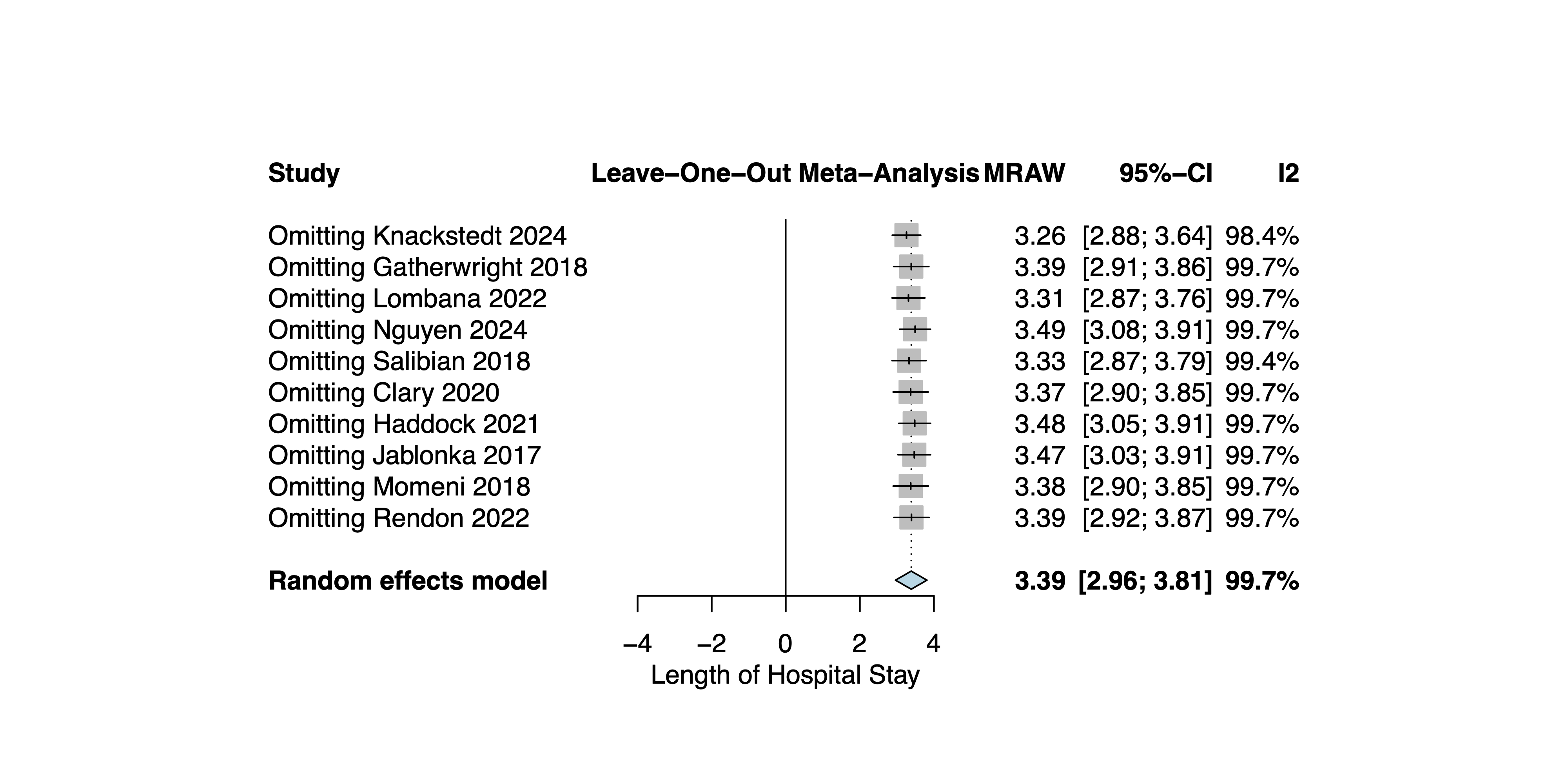

Supplement: Supplementary file 7 — Figure, Supplementary Digital Content 7. Sensitivity analysis of the length of hospital stays (single-arm meta-analysis). [file mmc7.zip › mmc7.png]

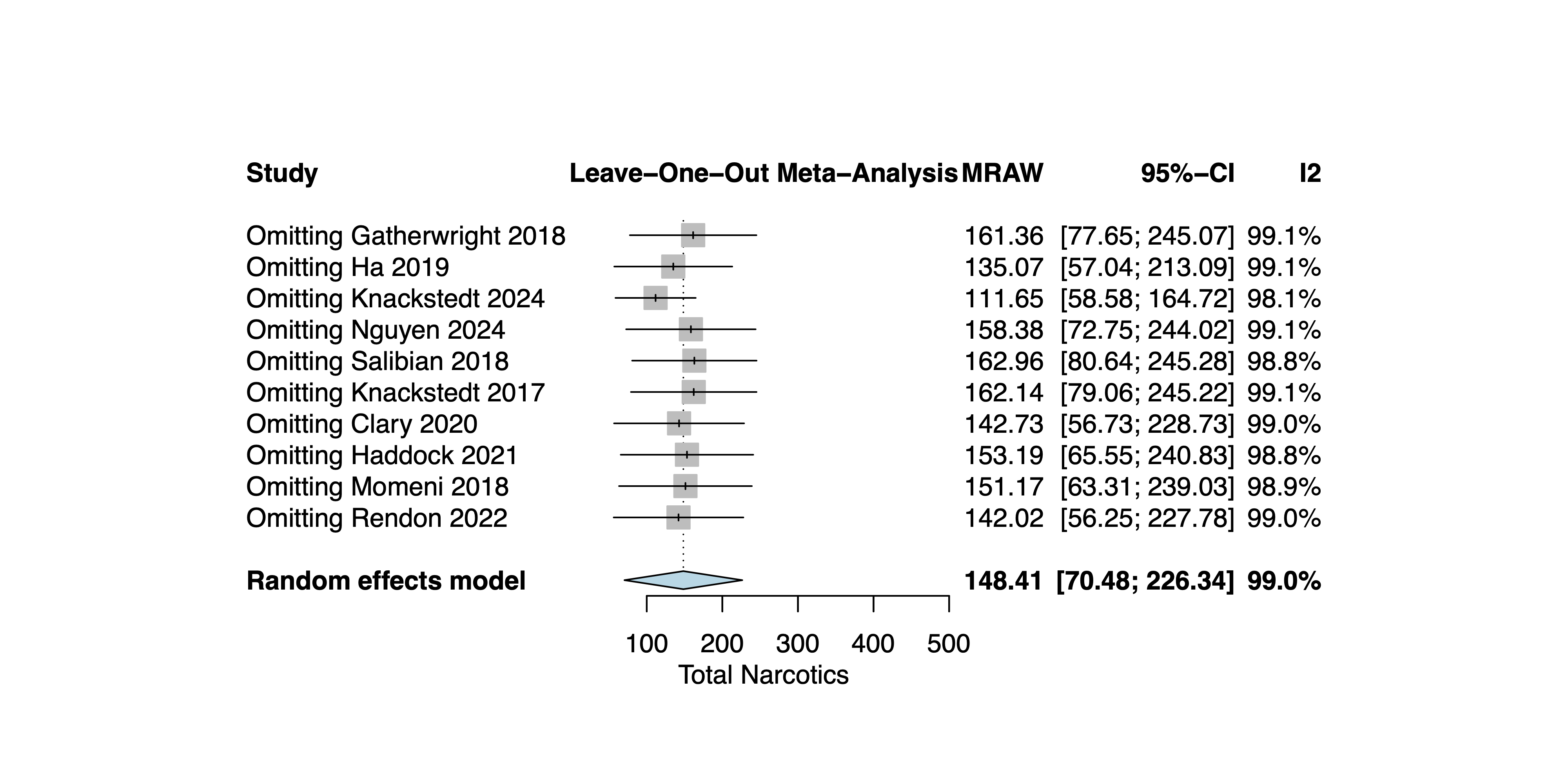

Supplement: Supplementary file 8 — Figure, Supplementary Digital Content 8. Sensitivity analysis of total narcotic use (single-arm meta-analysis). [file mmc8.zip › mmc8.png]

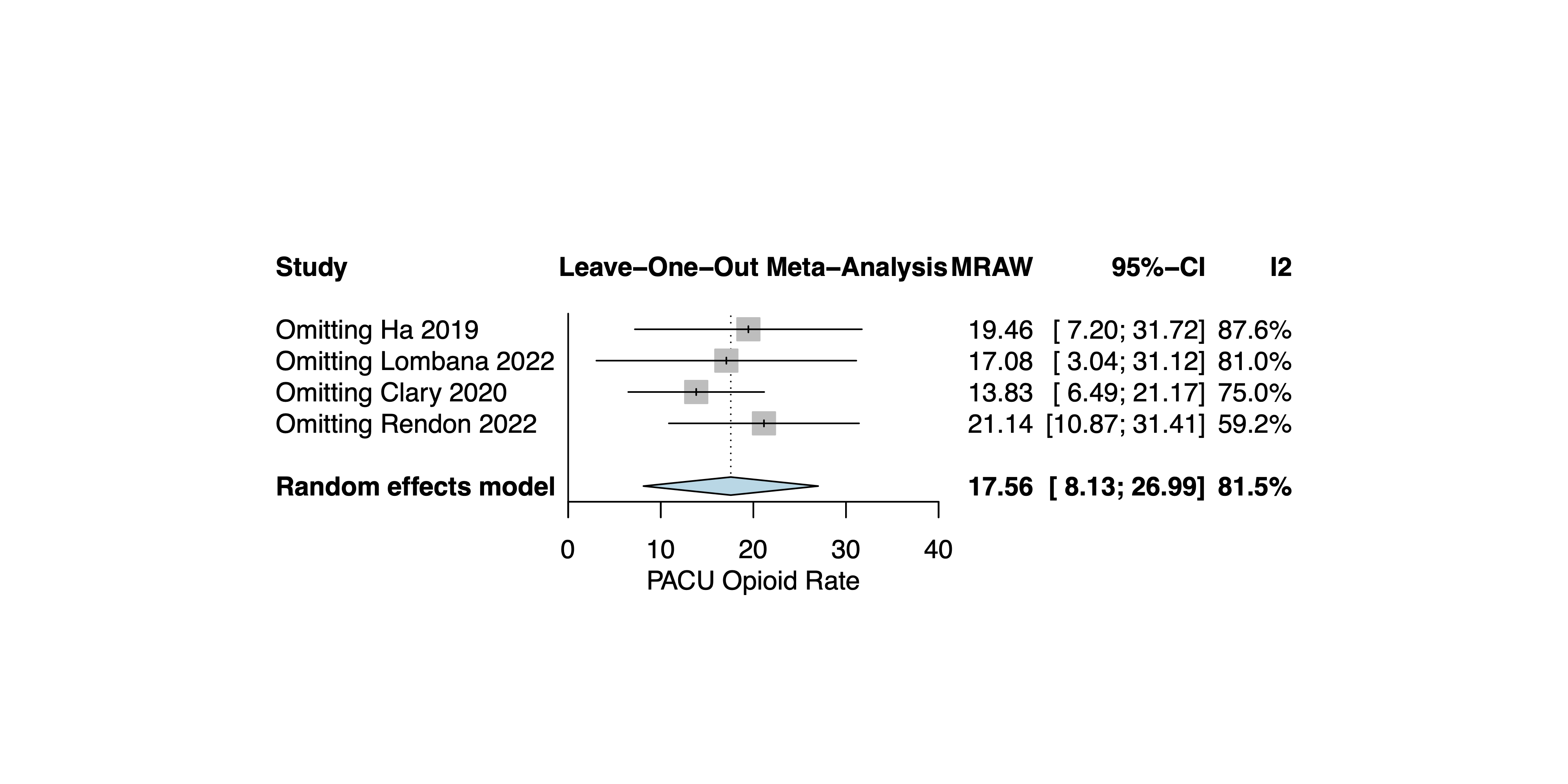

Supplement: Supplementary file 9 — Figure, Supplementary Digital Content 9. Sensitivity analysis of PACU opioid consumption (single-arm meta-analysis). [file mmc9.zip › mmc9.png]
